# Improving immunological tumor microenvironment using electro-hyperthermia followed by dendritic cell immunotherapy

**DOI:** 10.1186/s12885-015-1690-2

**Published:** 2015-10-15

**Authors:** Yuk-Wah Tsang, Cheng-Chung Huang, Kai-Lin Yang, Mau-Shin Chi, Hsin-Chien Chiang, Yu-Shan Wang, Gabor Andocs, Andras Szasz, Wen-Tyng Li, Kwan-Hwa Chi

**Affiliations:** 1 Department of Radiation Oncology, Chiayi Christian Hospital, Chiayi, Taiwan; 2 Department of Biomedical Engineering, Chung Yuan Christian University, Taoyuan City, Taiwan; 3 Department of Radiation Therapy and Oncology, Shin Kong Wu Ho-Su Memorial Hospital, Taipei, Taiwan; 4 Department of Radiological Sciences, Graduate School of Medicine and Pharmaceutical Sciences, University of Toyama, Toyama, Japan; 5 Department of Biotechnics, St. Istvan University, Budapest, Hungary; 6 Institute of Radiation Science and School of Medicine, National Yang-Ming University, Taipei, Taiwan

**Keywords:** Dendritic cells, Modulated electro-hyperthermia, Immunotherapy, Tumor microenvironment

## Abstract

**Background:**

The treatment of intratumoral dentritic cells (DCs) commonly fails because it cannot evoke immunity in a poor tumor microenvironment (TME). Modulated electro-hyperthermia (mEHT, trade-name: oncothermia) represents a significant technological advancement in the hyperthermia field, allowing the autofocusing of electromagnetic power on a cell membrane to generate massive apoptosis. This approach turns local immunogenic cancer cell death (apoptosis) into a systemic anti-tumor immune response and may be implemented by treatment with intratumoral DCs.

**Methods:**

The CT26 murine colorectal cancer model was used in this investigation. The inhibition of growth of the tumor and the systemic anti-tumor immune response were measured. The tumor was heated to a core temperature of 42 °C for 30 min. The matured synergetic DCs were intratumorally injected 24 h following mEHT was applied.

**Results:**

mEHT induced significant apoptosis and enhanced the release of heat shock protein70 (Hsp70) in CT26 tumors. Treatment with mEHT-DCs significantly inhibited CT26 tumor growth, relative to DCs alone or mEHT alone. The secondary tumor protection effect upon rechallenging was observed in mice that were treated with mEHT-DCs. Immunohistochemical staining of CD45 and F4/80 revealed that mEHT-DC treatment increased the number of leukocytes and macrophages. Most interestingly, mEHT also induced infiltrations of eosinophil, which has recently been reported to be an orchestrator of a specific T cell response. Cytotoxic T cell assay and ELISpot assay revealed a tumor-specific T cell activity.

**Conclusions:**

This study demonstrated that mEHT induces tumor cell apoptosis and enhances the release of Hsp70 from heated tumor cells, unlike conventional hyperthermia. mEHT can create a favorable tumor microenvironment for an immunological chain reaction that improves the success rate of intratumoral DC immunotherapy.

**Electronic supplementary material:**

The online version of this article (doi:10.1186/s12885-015-1690-2) contains supplementary material, which is available to authorized users.

## Background

The tumor microenvironment (TME) is an important factor in successful local treatment and in provoking a systemic immunological response in cancer patients [1]. Dendritic cells (DCs) that infiltrate the TME are responsible for the uptake of antigens *in situ* and maturation in the draining lymph nodes, and the provide the basis for effective anti-tumor T cell immune responses [2]. *In situ* DC-based cancer immunotherapy with radiotherapy has been utilized to treat cancer patients, but only a small number of tumor regressions have been observed [3]. A poor TME can cause DCs to differentiate into immunosuppressive regulatory DCs, which inhibit the effect of cytotoxic T cells activation and promote tumor progression [4]. The function of DCs is mainly positively affected by a microenvironment that contains fewer immune suppression factors, more immune potentiating factors and an immunogenic hub in the tumor site [5, 6]. This fact previously motivated us to develop a new strategy to improve the efficacy of *in situ* DC vaccination by adding combining heat shock protein (Hsp) [7] or by electro-gene therapy with cytokine [8]. How a therapy-induced anti-tumor immunity should be manipulated is not clearly known but immunogenic cancer cell death (ICD) has emerged as the most important sign of a favorable immunogenic TME [6, 9]. Only a favorable TME can provide the various important functional immunological cells and cytokines that are required for immunotherapy [10, 11].

Hyperthermia has been used in cancer therapy for decades. A branch of hyperthermia, known as modulated electro-hyperthermia [12–15] (mEHT – trade name: oncothermia) has been developed by the capacitive (impedance-based) coupling of 13.56 MHz amplitude-modulated radiofrequency energy at the tumor site [15]. The electric field energy may be selected and delivered to the malignant cells by exploiting the larger amount of ionic connective tissue around the tumor area, creating massive apoptosis at mild temperatures (≦42 °C) [14–17]. In Europe, mEHT has been successfully utilized in clinical treatment for over two decades [18–20]. Numerous retrospective studies of cancer patients have revealed that mEHT can treat a very wide range of tumor lesions and various types of tumor, demonstrating that the mEHT is a feasible option for treating cancer [14]. It is generally applied to treat various forms of malignant tumor, such as lung, liver, pancreas, brain, gastrointestinal, gynecological, and other such tumors. Qin et al. demonstrated that mEHT had an abscopal effect in experiments *in vivo* [21]. However, immature DCs that were used in Qin’s study may have increased the tolerance of antitumor immunity whereas mature DCs induce a strong antitumor immunity when they interact with cancer cells that are undergoing immunogenic cancer cell death (ICD) [22]. The combination of mEHT and the intra-tumoral injection of DCs may be able to provide a more sustained systemic immunity, enhancing the abscopal effect [23]. We hypothesize that mEHT is an ideal approach for changing the TME from immune-suppressive to immune-stimulatory. Mature DCs were utilized in this experiment to eliminate interference with the DC maturation process at tumor site and to observe the change in TME-induced mDC activation.

Although hyperthermia, combined with an intratumoral injection of DC, reportedly evokes systemic immunity, two applications of a moderately high temperature (43.7 °C for 1 h) are required to improve the induce an effective acquisition of antigens following three rounds of DCs treatment [24]. However, the temperature is not easily reached in clinical practice by conventional hyperthermia machine. Mild temperature hyperthermia (>42 °C) cannot generate massive apoptosis or cause a damage-associated molecular pattern (DAMP) in the tumor environment. The lack of release of tumor antigens from apoptotic tumor cells may dampen the effect of combined DCs and hyperthermia [21, 25–27]. mEHT has been demonstrated to induce massive apoptosis and a DAMP-related signal sequence in colorectal cancer xenografts at mild temperatures [28]. A favorable anti-tumor immune microenvironment at a mild temperature may be more effective in promoting an immunological cell death response [28]. For the above reasons, this work proposes that combining intratumoral DCs at mild temperature with mEHT may be more effective in generating tumor cell apoptosis and DAMP and in providing a favorable immunological environment eliciting specific immunity. The data obtained herein evidence that mEHT may change TME immune phenotypes, including infiltrated leukocytes and eosinophils, and be feasibly combined with intra-tumoral DCs immunotherapy.

## Methods

### Cell lines and mice

CT26, a murine colon carcinoma cell line that is derived from a BALB/c mouse, was purchased from the Culture Collection and Research Center (Hsinchu,Taiwan), where fresh batches are thawed every year. CT26 cells were maintained in Dulbecco’s modified Eagle medium (DMEM) was supplemented with 10 % fetal bovine serum (FBS), 100 ng/ml of streptomycin, and 100 U/ml of penicillin (Invitrogen). Female BALB/c mice were obtained from the National Science Council Animal Center, Taipei, Taiwan, and were used at between 6 and 8 weeks of age. This study was approved by the Institutional Animal Care and Use Committee of the Shin Kong Wu Ho-Su Memorial Hospital (Approval No. 0990827008).

### Modulated electro-hyperthermia treatment (mEHT)

Electromagnetic heating was conducted using capacitive-coupling with an amplitude-modulated 13.56-MHz radiofrequency (LabEHY, Oncotherm, Germany). The mEHT technical details of the method can be found elsewhere [29]. An *in vitro* heating model was established in an electrode chamber (LabEHY in vitro applicator), which was heated to 42 °C for 30 min at a mean power of 8 ~ 9 W. The cells were placed in a chamber with a culture medium at 42 °C for 30 min. Tumor implants in the right femoral area of BALB/c mice were placed in the parallel electric condenser of the heating circuit, as described elsewhere [28]. The treatment groups were givena single shot of mEHT for 30 min at a mean power of 1.5 W under 100 mg/kg Ketamine and 10 mg/kg Xylazine anesthesia. Intratumoral temperature was maintained at ~ 42 °C on the treated side of each mice, as measured using optical sensors (Luxtron FOT Lab Kit, LumaSense Technologies, Inc., California, USA). The subcutaneous temperature underneath the electrode was maintained at 38 ~ 40 °C.

### Apoptosis assay

Water bath-treated and mEHT-treated CT26 cells were cultured for 24 h, then trypsinized, and washed twice with PBS. Apoptosis was verified using an Annexin V Apoptosis Kit (BD Pharmingen), following the manufacturer’s instructions. Briefly, tumor cells were washed three times with PBS; then, some cells were analyzed immediately for apoptosis using Annexin V/PI staining. Washed cells were supplemented with 1 % BSA and then stained directly with 10 μL of PI and 2.5 μL Annexin V-FITC, following the addition of 222.5 μL of binding buffer. Immediately after 10 min of incubation in the dark on ice, the cells were analyzed by flow cytometry. The percentage of positive cells was determined using a FACSCalibur cytometer and Cell Quest Pro software (Becton Dickinson, Mountain View, CA).

### Western blot analysis

For protein analysis, the water bath-treated control and mEHT-treated CT26 cells were lysed for 5 min at room temperature in a buffer of 150 mM NaCl, 50 mM Tris (pH 8.0), 5 mM EDTA, 1 % (v/v) Nonidet p-40, 1 mM phenylmethylsulfonyl fluoride, 20 μg/mL aprotinin, and 25 μg/mL leupeptin (Sigma). The total protein concentration was measured using the Bio-Rad protein assay reagent. Cell lysates (100 μg) were electrophoresed on a 12 % polyacrylamide gel, transferred onto an Immobilon-P PVDF membrane (Millipore, Bedford, MA), and blocked in PBS-Tween 20 and 10 % nonfat milk for 2 h at room temperature. The filter was incubated with specific antibodies to anti-Hsp70 (Santa Cruz Biotechnology, Santa Cruz, CA) and anti-HMGB-1 (Abcam, Cambridge, MA, USA) for 2 h at room temperature in PBS-0.05 % Tween 20 that contained 5 % nonfat milk, followed by 1 h incubation at room temperature with horseradish peroxidase-conjugated secondary antibodies (Jackson ImmunoResearch Laboratories, West Grove, PA) in the same buffer. Blots were developed using a chemiluminescent detection system (ECL; GE Life Science, Buckinghamshire, UK).

### Hsp70 release assay

mEHT treated CT26 cells were cultured for 24 h. The culture supernatants were harvested and Hsp70 was measured by using an enzyme-linked immunosorbent assay (ELISA) (Enzo Life Sciences, Farmingdale, USA). A Multiskan Plus (Thermo Scientific, Hudson, NH, USA) was utilized to measure absorbance at 450 nm.

### Generation of bone marrow-derived dendritic cells

Bone marrow-derived DCs (BM-DCs) were produced as described elsewhere [9]. Briefly, BM-DCs were isolated from BALB/c mice by culturing red blood cell-depleted BM cells in a complete medium (RPMI 1640 that was supplemented with 10 % FBS, L-glutamine, and 5 mM 2-mercaptoethanol) that contained 20 ng/ml of recombinant mouse GM-CSF (Peprotech, Rocky Hill, NJ, USA) at 37 °C in a humidified atmosphere with 5 % CO_2_ and fed every third day with a medium that contained fresh GM-CSF. On day nine of the culture, the DCs were mixed with 10 μg/ml AH1 (SPSYVYHQF) that had been manufactured at 95 % purity by AnaSpec (Fremont, CA) and 50 μg/ml Hsp70 which was prepared in our laboratory as described elsewhere [7] for 24 h. On day ten of the culture, non-adherent cells were harvested, washed once in a complete medium, and examined to evaluate the expression of the DC surface markers (MHC class II molecule I-A^d^/I-E^d^, CD80 (B7-1), CD86 (B7-2), CD11c, and DEC205). The BM-DCs (5–10 × 10^5^) were stained with 50 μL of FITC-conjugated antibodies in phosphate-buffered saline (PBS) that contained 1 % bovine serum albumin (BSA) and 0.1 % azide, which was also used as the washing buffer, before being subjected to fluorescence-activated cell sorting (FACS) analysis using a FASCalibur flow cytometer (BD Bioscience, San Diego, California, USA). Cells were stained with the corresponding isotype-matched control IgG (BD Pharmingen, San Diego, CA, USA). Endocytic activity was quantifiedby incubating cells for 2 h with FITC-dextran (100 μg/ml) (Sigma) at 4 °C or 37 °C. Cells were washed extensively with PBS, before being subjected to FACS analysis. Non-specific binding of FITC-dextran to the cell surface was measured by incubating the cells at 4 °C [30]. The percentage of positive cells was obtained using a FACSCalibur cytometer and Cell Quest Pro software (Becton Dickinson, Mountain View, CA).

### Animal study

On day zero, the right femoral areas of BALB/c mice were injected subcutaneously with 5 × 10^5^ CT26 tumor cells. On day 14 following injection, the mice received local mEHT treatment (as described above), and then, on the following day, 5 × 10^5^ syngeneic DCs or PBS in 25 μL were injected into the right femoral tumor area. Each group comprised ten mice. Sampling was carried out 48 h following treatment, using three mice in each group. Each excised tumor was fixed in 10 % formalin, dehydrated, and embedded in paraffin wax (FFPE). The sizes of the tumors in the other seven mice in each group were measured at least three times weekly: length (L) and width (W) were recorded and the tumor volumes were calculated as L × W^2^/2. To evaluate whether specific immunologic memory responses were generated in mice that bore CT26 tumor cells, the mice were re-challenged with 1 × 10^5^ tumor cells in the other flank 30 days following the first tumor inoculation [9]. The mice were examined three times weekly to evaluate tumor development for 30 days after tumor cell transplantation or the first inoculated tumor growth until the tumor was more than 2 cm in diameter.

### Cytotoxicity T lymphocyte (CTL) assay

On day 30 following tumor injection, the mice were killed and their spleens harvested. Erythrocyte-depleted splenocytes (1 × 10^6^ cells/ml) were cultured for five days *in vitro* using mitomycin C-treated CT26 tumor cells (1 × 10^6^ cells/ml) in 24-well plates, during which time 50 IU/ml of recombinant human IL-2 (Proleukin; Novartis Pharmaceuticals, East Hanover, NJ) was added daily. On day five, the cells were collected; dead cells were removed on a density gradient, and the viable cells were tested to evaluate specific cytotoxicity using LDH-release assay (Promega, Madison, WI, USA). The percentage-specific cytotoxicity was calculated as 100 x [(experimental release – spontaneous release)/(maximal release – spontaneous release)].

### Enzyme-linked immunosorbent spot (ELISPOT) assay

The ELISPOT assay was conducted using a Mouse IFN-γ Development Module kit (R&D System), following the manufacturer’s instructions. Splenocytes were prepared as described for use in the CTL reactions. The harvested splenocytes (1 × 10^5^ in 100 μL) were then mixed with 100 μL of CT26 tumor lysate (50 μg of protein/ml) in each well of a 96-well filtration plate (MultiscreenTM HTS) that had been previously coated with capture antibodies (1:60 dilution). The negative controls were the medium alone and the splenocytes alone and the positive control was splenocytes plus 20 μg/ml of Con A. After incubation overnight at 37 °C, color was developed using the streptavidin-alkaline peroxidase and BCIP/NBT that was provided in the ELISPOT kits. The spots were counted visually under a dissection microscope; the numbers of spots in the test samples (splenocytes + tumor lysate), spots obtained using splenocytes alone, and spots obtained using medium alone were calculated.

### Immunohistochemistry and Luna stain

To conduct immunohistochemical studies, the tumor was resected and fixed in 10 % formalin for 24 h. To stain the sections immunohistochemically, paraffin sections were deparaffinized in xylene and rehydrated in a graded alcohol series, treated with 3 % H_2_O_2_ for 10 min, and boiled in a citrate buffer (pH 6) for 30 min (anti-F4/80 antibody, Bioss bs-7058R, anti-CD45 antibody, Bioss bs-0522R), before immunoblock (Bio TnA, TAHC03) was applied to prevent non-specific binding for 60 min at room temperature. The sections were incubated with rabbit anti-F4/80 antibody (diluted 1:100) and anti-CD45 antibody (diluted 1:100) for one hour at 37 °C, and analyzed by Mouse/Rabbit Probe HRP labeling (BioTnA, TAHC03) for 30 min at room temperature. Peroxidase activity was developed in a diaminobenzidine- H_2_O_2_ solution (Bio TnA, TAHC03) for 10 min at room temperature. The sections were then counterstained with hematoxylin. All stained slides were examined by two pathologists who were blind to the treatment group data. The percentage of positively stained cell membranes or cytoplasm was obtained by microscopically examining the entire tissue at high magnification (×400). The numbers of positive cells was calculated in ten fields. The Luna protocol was performed as described elsewhere with slight modifications [31]. The sections were immersed in working Hematoxylin-Biebrich (Sigma, Cat # H-3136 and Acros, CI 26905, respectively) scarlet solution (for five minutes), and then dipped (∼8x) in 1 % acid alcohol and rinsed in tap water. The sections were then dipped (∼5x) in lithium carbonate solution until they turned blue and washed in running tap water (for two minutes). The numbers of eosinophil on the stained slide were calculated in ten fields (x400).

### Statistical analysis

All results were compared using an unpaired *t* test (two-tailed) or one-way ANOVA. Differences were considered statistically significant at a *P* value of less than 0.05

## Results

### mEHT induced more apoptotic cell death than water bath-induced hyperthermia in CT26 cells

**Fig. 1:**
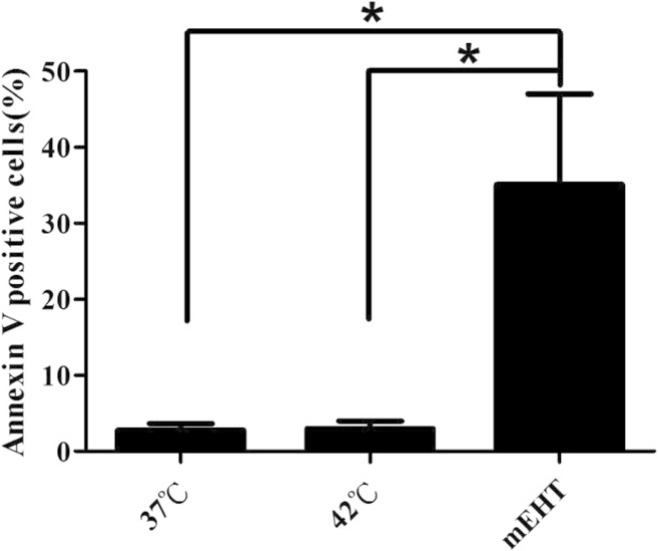
Apoptosis in mEHT-treated CT26 cells. One and a half million CT26 cells were heated to 42 °C for 30 min using LabEHY or a water bath (control). The apoptosis of CT26 cells after 24 h of hyperthermia treatment was analyzed using Annexin-V assay. (*, *p* < 0.05; *n* = 3)

The apoptotic efficacy of hyperthermia that was induced by a water bath or mEHT in CT26 cells was evaluated using an apoptosis assay kit. mEHT treatment significantly increased the percentage of apoptotic cells (35.10 %) above that of the 37 °C control (2.76 %) or the water bath control (2.98 %, *p* < 0.05) (Fig. 1). This result suggests that mEHT increased the susceptibility of CT26 cells to apoptosis, whereas the water bath did not.

### mEHT promoted the generation and release of Hsp70 in CT26 cells

**Fig. 2:**
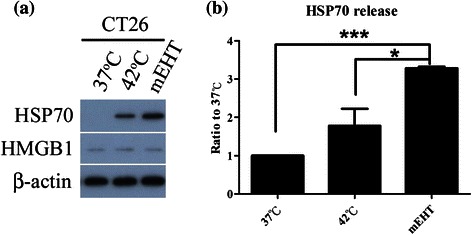
Expression and release of Hsp70 following hyperthermia treatment. One and a half million CT26 cells were heated to 42 °C for 30 min using LabEHY or a water bath (control). **a** After 24 h of incubation, cells were harvested for western blot analysis of Hsp70 or HMGB1 protein in lysates of CT26 (control) (37 °C), a water bath (control) (42 °C) or mEHT. β-actin was used as an internal control. **b** After 24 h of incubation, supernatants were harvested and concentration of Hsp70 was measured using ELISA (*n* = 3). Data are presented as mean +/− SD from three independent experiments. * indicates *p* < 0.05

Since the expression of Hsp70 is a characteristic of hyperthermia treatment, the intracellular amount of Hsp70 and the release of Hsp70 following mEHT and water bath-induced hyperthermia were investigated. Whole-cell extracts were prepared for western blot against Hsp70. The released Hsp70 was collected from the cell culture supernatant and assayed by ELISA. Both water bath and mEHT treatment increased the expression of Hsp70 in CT26 cells (Fig. 2a). Interestingly, only mEHT significantly increased the release of Hsp70 for hyperthermia (Fig. 2b). Another danger signal, high mobility group box 1 (HMGB1) protein, did not show any difference in mEHT as compared to water bath-induced hyperthermia (Fig. 2a). Those data revealed that mEHT induced greater Hsp70 responses at a cellular level than did water bath-induced hyperthermia.

### AH1 and Hsp70 induce maturation of BM-DCs

A CT26-derived epitope (AH1) [32] that was presented by H-2Dd and Hsp70 was used as an antigen to stimulate DC maturation, as described elsewhere [7]. The maturation of DCs was quantified from the expression of specific cell surface markers, and the relevant data were collected from three independent experiments. Additional file 1: Figure A reveals that a high proportion of the cells were DC-positive for CD11c (MFI: 51.20 ± 20.04), MHC class II (MFI: 74.43 ± 15.62) and CD80 (MFI: 32.45 ± 7.91) antigens, and that AH1 and Hsp70 upregulated the expression of the mouse DC maturation markers MHC class II (MFI: 283.33 ± 119.30) and CD80 (MFI: 108.79 ± 26.60) on BM-DCs. Immature DCs exhibit potent endocytotic activity, which declines upon maturation. The endocytotic activity of the BM-DCs was evaluated before and after AH1 and Hsp70 treatment by measuring the phagocytosis of mannose-receptor-mediated FITC-Dextran. The uptake was significantly lower (*P* < 0.05) following AH1 and Hsp70 treatment than before (35.2 % ±11.4 % versus 85.9 % ±7.9 %) (Additional file 1: Figure B).

### Combination of both local and systemic mEHT induced anti-tumor effect of DC therapy *in vivo*

**Fig. 3:**
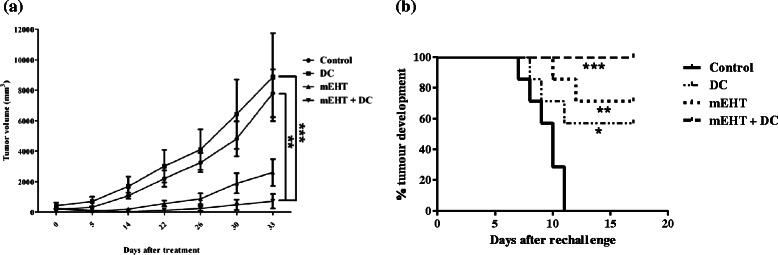
Inhibition of tumor growth and rechallenge inoculation. **a** Mice in different groups were injected with 5 × 10^5^ CT26 tumor cells (subcutaneously) in right femoral area on day zero and treated with mEHT on day 14, before receiving DC injection on day 15. Data obtained from each mouse after tumor-cell inoculation (*n* = 7) were plotted. **b** A secondary rechallenge with CT26 tumor cells was administered to mice 30 days after first injection with DC alone or following mEHT or mEHT-DC therapy. Contra-lateral flanks of mice in treated groups and untreated control BALB/c mice were inoculated subcutaneously (1 × 10^5^ parental CT26 cells). Percentage of mice that developed tumors at contra-lateral site was obtained using Kaplan–Meier method. (*n* = 7 mice per group.)

First, whether local mEHT-DC immunotherapy could induce an either local therapeutic effect or systemic antitumor effects was tested outside of the treatment field in tumor-bearing mice. Based on the hypothesis that the indirect antitumor effects of DC are mediated by its ability to promote cross-priming and, therefore, trigger systemic antitumor immunity, mEHT was administered to mice before DC injection to change the TME and facilitate this process. Tumors were treated to quantify the direct therapeutic effect of mEHT-DC therapy (Fig. 3a) by injecting DCs into the tumor site 24 h following mEHT treatment. Two days later, three tumors were removed for immunohistochemical staining, and the sizes of the tumors in the rest of the mice were measured every two or three days weekly. mEHT-DC therapy significantly delayed local tumor growth (Fig. 3a), and complete tumor regression was observed in five out of seven mice in this group. Interestingly, mEHT treatment alone also caused a significant growth delay relative to the control mice or mice treated with DC alone (*P* < 0.05); complete tumor regression was observed in two out of seven mice in the mEHT-treatment-alone group.

### mEHT-DC treatment improves immunogenicity

To evaluate both the direct and systemic effects of mEHT-DC therapy, a rechallenge model was utilized in which mice had one tumor first and were inoculated with a secondary tumor one month later. All mice that were originally treated with mEHT-DC showed complete rejection of a secondary rechallenge (Fig. 3b). In contrast, five out of seven mEHT-treated mice and four out of seven DC-treated mice rejected the rechallenge of CT26 cells. All of the untreated control mice grew the rechallenge tumor within 12 days. This result indicates that mEHT-DC treatment induces an effective antitumor memory response.

### mEHT-DC therapy increases number of tumor-infiltrating leukocytes

**Fig. 4:**
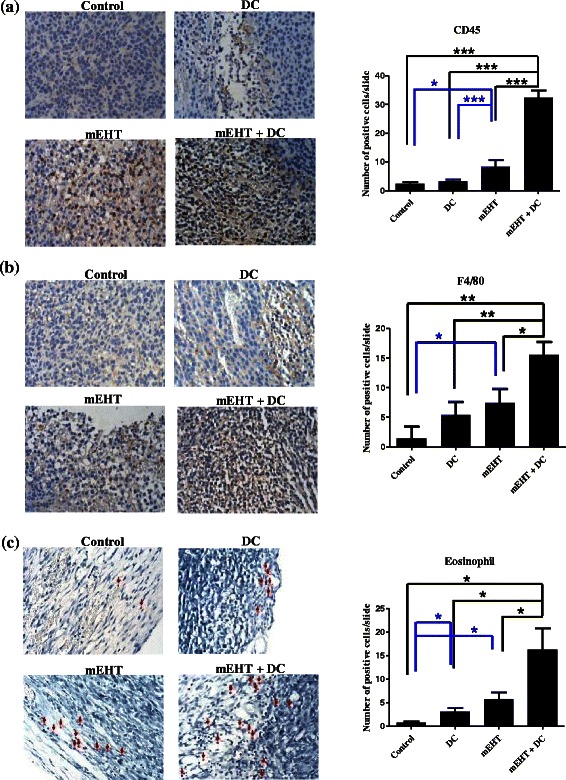
Areas of tumor infiltrated by immune cells after mEHT and DC treatment. Representative images of immunohistochemical straining revealed that quantities of CD45 (**a**) and F4/80 (**b**) were increased in tumors that were treated with DC and mEHT, either alone or in combination. Proportion of positive cells in one field that was randomly selected from ten fields, was calculated. **c** Amount of eosinophil increased significantly upon treatment of tumor; error bars represent standard errors. (***) *P* < 0.001 (t-test) relative to control. EH: electro-hyperethermia

To elucidate the anti-tumor immune response that is generated by treatment with mEHT and DC, tumors from the treated and control mice were extracted and processed for immunohistochemical analysis two days after treatment. Tumor-infiltrating CD45^+^ leukocytes and F4/80^+^ macrophages were identified. The presence of tumor-infiltrating CD45^+^ leukocytes was significantly higher in mice that had been treated with mEHT-DC than in those that had been treated with mEHT alone, those that had been treated with DC alone and the control mice. mEHT alone significantly increased the infiltration of CD45^+^ leukocytes over that achieved using DC alone or evident in the control mice (Fig. 4a). An increase in tumor-infiltrating F4/80^+^ macrophages was observed following the combined administration of mEHT/DC (Fig. 4b). This result demonstrates that mEHT induced an inflammatory environment and that combined treatment strengthened this inflammatory response. In addition, tumor-associated eosinophilia is frequently observed in some cancers. Several studies have shown that eosinophils are attracted into tumors by chemotactic factors [33, 34]. However, the role of infiltrating eosinophils in tumor is unclear. Recently, a published study demonstrated that activated eosinophils caused substantial changes in the tumor microenvironment, including the polarization of macrophages and normalization of the tumor vasculature, which are known to promote tumor regression [35]. Therefore, eosinophils were examined herein by Luna staining. According to Fig. 4c, the presence of tumor-infiltrating eosinophils was significantly higher in the mice that were treated with mEHT-DC than in the other groups. This result demonstrates that mEHT recruited eosinophils into the tumor tissue and might have induced an inflammatory response.

### mEHT-DC therapy induced cytotoxic antigen-specific CD8^+^ effector cells

**Fig. 5:**
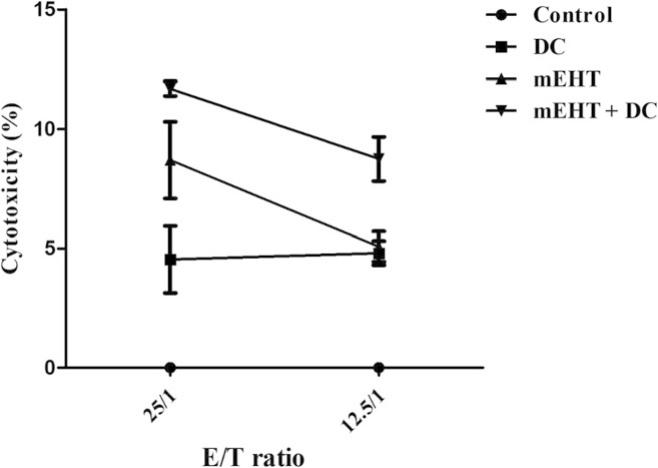
Tumor-specific CTLs. Mice in various groups were injected with 5 × 10^5^ CT26 tumor cells subcutaneously on day zero and with mEHT on day 14, before being given DC injection on day 15. On day 30 following tumor injection, splenocytes were harvested for CTL assay. Cytotoxic activity of splenocytes was determined by LDH-release assay at various effector/target cells (E/T) ratios

Seven days following DC injection, two other mice were sacrificed and the induction of AH1-specific IFN-γ–producing CD8^+^ cells was tested after the T cells were restimulated with the AH1 recombinant protein in an ELISPOT assay. The cytolytic activity of responding T cells was tested directly on AH1-positive CT26 target cells in an LDH-release assay. As shown in Fig. 5, specific cytotoxicity was significantly higher in mice that were co-treated with mEHT and DC than in those that were treated with DCs alone, those that were treated mEHT alone or the control mice after splenocytes had been stimulated with AH1 recombinant protein (*P* < 0.05). The lytic activity of the cultured spleen cells correlated strongly with the degree of inhibition of tumor growth in each group, as presented in Fig. 3. mEHT-DC therapy significantly increased the T cell-mediated cytotoxicity over that observed in control mice, while DC alone had no significant effect.

**Fig. 6:**
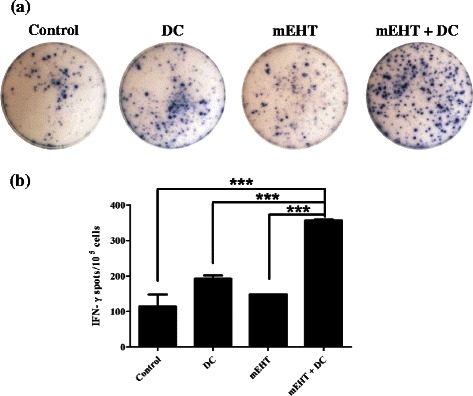
ELISpot assays. **a** Representative results of ELISpot assay of mice splenocytes that were pulsed with AH1. Top two rows, 2 × 10^5^ splenocytes/well; bottom two rows, 10^5^ splenocytes/well. PC: positive control, splenocytes treated with ConA (5 mg/ml) for 24 h. NC: negative control, splenocytes treated with BSA for 24 h. **b** Numbers of IFN-γ-secreting T-cells in DC-treated and mEHT + DC-treated mice significantly exceeded those in mice treated with mEHT alone and untreated control mice. Error bars represent standard errors. (***) *P* < 0.001 (t-test) relative to control. EH: electro-hyperethermia

Figure 6a presents representative results of the ELISPOT assays of cells that were pulsed with AH1 recombinant protein. The number of IFN-γ-secreting CD8^+^ T-cells in mice that were co-treated with mEHT and DC significantly exceeded the corresponding numbers in mice that were treated with DCs alone, mEHT alone or the control (Fig. 6b; *P* < 0.001, one-way ANOVA). These results indicate that the intra-tumoral injection of DCs combined with mEHT therapy induced stronger antigen-specific immunity than did treatment with DCs alone or mEHT alone.

## Discussion

This study found that the combination of mEHT at a clinical achievable temperature (42 °C) with the intra-tumoral injection of DCs not only elicits a local antitumor response but also induces a systemic anti-tumor immune response. A tumor-specific T cell response was evoked. The ability of mEHT to induce apoptosis in a high percentage of tumor cells and enhance the release of Hsp70 is believed to be a key contributor to the tumor-specific immune response. In our previous study, co-injection of rHsp70 and DCs was found to turn radiation-induced local apoptosis into a systemic anti-tumor immune response. Co-injection of rHsp70 and DCs into the irradiated tumor site caused a more potent anti-tumor immune response than did the injection of DCs alone [7]. In this study, mEHT caused a heated tumor to release more Hsp70 into extracellular spaces than did other hyperthermic methods. The release of Hsp70 served as a danger signal that made the TME a more immunologically responsive milieu, in which infiltration by eosinophils.

Mukhopadhaya et al. reported that localized hyperthermia, following by heating in a water bath at 43.5 °C, combined with intratumoral DC induced systemic antitumor immunity [24]. Heat treatment (>43.5 °C) induces both apoptosis and necrosis and the release of Hsp70 from cancer cells and triggers DC activation. However, several points must be addressed. First, heating by a water bath cannot be used in clinical practice. Heating a localized tumor to 43 °C using conventional hyperthermia machines is very difficult. In the experiment in this study, hyperthermia that was generated using a water bath at 42 °C induced only a limited apoptotic or necrotic effect in cancer cells. Another radiofrequency machine, operated at 42 °C for 30 min, also failed to have cause significant apoptosis in cells (unpublished data). Only mEHT induced significant apoptotic cell death at 42 °C for 30 min. Accordingly, the combination of mEHT with intratumoral DC immunotherapy should be clinically feasible. We have previously reported that the intratumoral injection of immature DCs into the irradiated tumor (RT-DC treatment) elicits tumor-specific immunity in hepatocellular carcinoma patients [3]. The present study recommends a future clinical trial of a combination of mEHT, intra-tumoral injection of DCs and radiotherapy.

The use of mEHT treatment as adjuvant for DC therapy to induce immunogenic apoptotic cell death by has recently been examined [21, 36]. However, the incubation of stressed, apoptotic tumor cells with syngeneic DCs is generally not strong enough to generate protective immunity, suggesting that the uptake of apoptotic cells by DCs alone may not be sufficiently efficient to activate an immune response, as described by others [37]. In this study, DC alone was not effective in inducing an immune response. The suppressive monocytes that are formed by a CT26 tumor have an important role in general immunosuppression [38]. These suppressive monocytes may inhibit the function of therapeutic DC. Therefore, the secondary signals that are required to activate DC function are induced by danger molecular pattern proteins, such as calreticulin, HSPs, and the HMGB1 [39]. mEHT provides danger molecular pattern proteins and induces inflammatory signals in tumor microenvironments [28]. Apoptotic cells cannot effectively activate DC activation in the absence of inflammatory signals [37, 40, 41]. Candido et al. [42] found that intratumoral administration of DC can partially inhibit the growth of an established tumor, but the co-administration of inflammation cytokine (TNF-α) strengthens the DC-mediated anti-tumor effect, consistent with our observations.

mEHT reversed the immunosuppressive microenvironment to an inflammatory environment and induced a DC-mediated anti-tumor immune response [21]. The inflammatory environment herein was evidenced by the infiltration of CD45^+^ cells and F4/80^+^ cells (Fig. 4). Tumor-infiltrating CD45RO^+^-cell density has been identified as a prognostic biomarker that is associated with the longer survival of colorectal cancer patients [43]. mEHT improves the expression of this prognostic biomarker.

Changing the TME into an inflammatory environment is important in ensuring that the DCs function in a manner that triggers systemic immunity and that the effector T cells adequately kill tumors. Although several studies have shown that DC therapy induces immune responses in cancer patients, few reports have demonstrated any clinical benefit of DC treatment perhaps because of the inhibitory effect of the TME. To improve the efficacy of DC therapy, the immunosuppressive status of the TME must be reprogrammed [44]. Immune checkpoint blockage therapy was recently shown to have a promising therapeutic effect on cancer patients [45]. The combination of positive immunotherapy with an anti-negative immune checkpoint inhibitor is a synergistic strategy [46]. The success of immune check point therapy demonstrates that elimination of the inhibitory pathways that block effective antitumor T cell responses is important in cancer therapy. However, the removal of inhibitory pathways from the immune system does not suffice to cure all of a cancer: increasing the number of immunopotentiation tumor-infiltrating cells, including CD45-positive leukocytes [46] and eosinophils, may be equally important [35]. However, a tumor tissue that lacks immunological markers may represent a nonimmunologic TME. The TME must be turned into an immunologic TME before DC therapy can activate T cells. Reciprocally, active T cells depend on an immunological TME to have a positive clinical effect. Therefore, combined treatment with mEHT and DCs should help to establish OR an immunogenic TME that has a clinical benefit for patients, independently of whether the preexisting tumor was immunogenic or nonimmunologic. Owing to the clinical feasibility of mEHT, this result suggests future clinical applications.

## Conclusion

In summary, mEHT is more effective in combined immunotherapy than is conventional hyperthermia in a clinically viable temperature range. Hyperthermia-induced local tumor apoptosis and release of Hsp70 can be translated into a systemic anti-tumor immune response. Since mEHT treatment alone is not sufficiently powerful to generate a systemic specific anti-tumor immune response, the best treatment strategy may be to increase the immunogenicity of non- or weakly immunogenic tumor cells by promoting their DC function. Further clinical investigation will be initiated accordingly.

## Additional file

Additional file 1: Figure A. (a) Representative flow cytometric analysis on immature and mature DCs. The purple histograms represent the isotype-matched control, and the red line histograms represent staining with specific antibodies. *MFI represents the mean fluorescence intensity, which are expressed on the right upper corner of each histogram. Error bars represent standard errors. (b) Histogram plot of MFI. (*) P < 0.05, (**) P < 0.01 (t-test) compared with the immature DC. **Figure B.** Endocytotic activity was measured by flow cytometry in DCs generated from bone marrow cultured for 9 d with 20 ng/ml of GM-CSF; mDCs obtained from iDCs were incubated with 10 g/mL of AH1 and 50 g/mL of Hsp70 for 24 h and reacted with 100 mg/mL of FITC-Dextran at 4°C (purple) or 37 °C (green line) for 2 h before analysis. This result was represented from one of three independent experiments. (DOCX 608 kb)

## Abbreviation

TME: Tumor microenvironment
DCs: Dendritic cells
mEHT: Modulated Electro-hyperthermia
Hsp70: Heat shock protein70
ICD: Immunogenic cancer cell death
DAMP: Damage-associated molecular pattern
CTL: Cytotoxic T lymphocyte
ELISPOT: Enzyme-linked immunosorbent spot
