# Supplementary material for: Improving immunological tumor microenvironment using electro-hyperthermia followed by dendritic cell immunotherapy
Source: BMC Cancer. 2015 Oct 15;15:708. doi: 10.1186/s12885-015-1690-2 (PMC4608323; doi:10.1186/s12885-015-1690-2)
Supplement: Additional file 1: Figure A. — (a) Representative flow cytometric analysis on immature and mature DCs. The purple histograms represent the isotype-matched control, and the red line histograms represent staining with specific antibodies. *MFI represents the mean fluorescence intensity, which are expressed on the right upper corner of each histogram. Error bars represent standard errors. (b) Histogram plot of MFI. (*) P < 0.05, (**) P < 0.01 (t-test) compared with the immature DC. Figure B. Endocytotic activity was measured by flow cytometry in DCs generated from bone marrow cultured for 9 d with 20 ng/ml of GM-CSF; mDCs obtained from iDCs were incubated with 10 g/mL of AH1 and 50 g/mL of Hsp70 for 24 h and reacted with 100 mg/mL of FITC-Dextran at 4°C (purple) or 37 °C (green line) for 2 h before analysis. This result was represented from one of three independent experiments. (DOCX 608 kb) [file 12885_2015_1690_MOESM1_ESM.docx]

(a)

| (b)  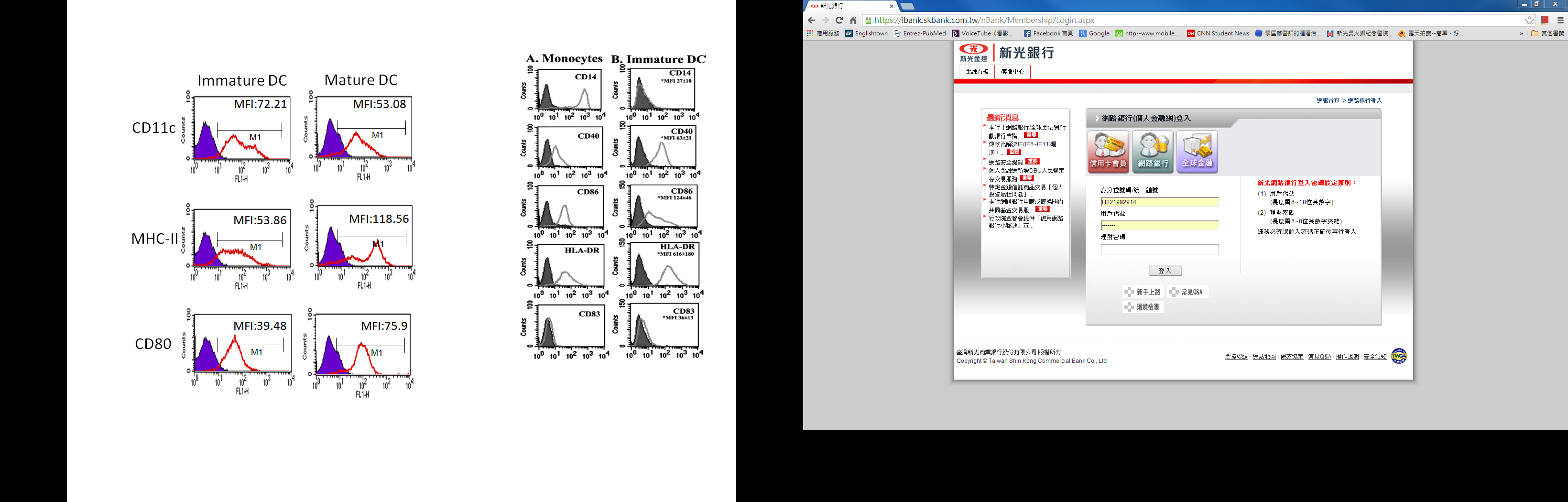 |
| --- |
| **Supplementary Figure A** (a) Representative flow cytometric analysis on immature and mature DCs. The purple histograms represent the isotype-matched control, and the red line histograms represent staining with specific antibodies. *MFI represents the mean fluorescence intensity, which are expressed on the right upper corner of each histogram. Error bars represent standard errors. (b) Histogram plot of MFI. (*) P < 0.05, (**) P < 0.01 (t-test) compared with the immature DC. |


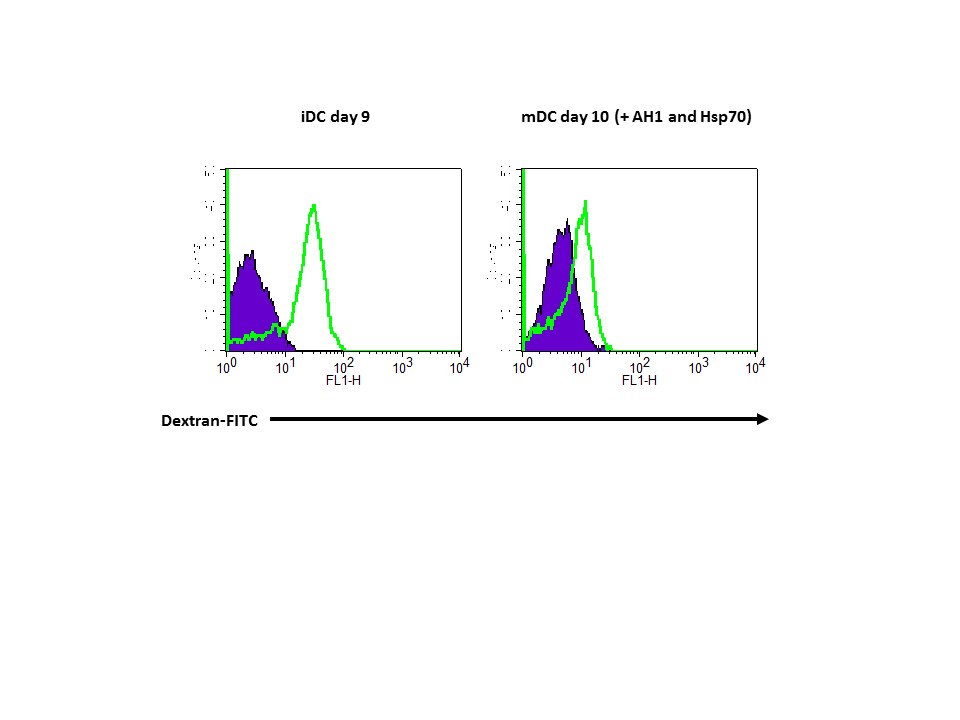


**Supplementary Figure B** Endocytotic activity was measured by flow cytometry in DCs generated from bone marrow cultured for 9 d with 20 ng/ml of GM-CSF; mDCs obtained from iDCs were incubated with 10 μg/mL of AH1 and 50 μg/mL of Hsp70 for 24 h and reacted with 100 mg/mL of FITC-Dextran at 4°C (purple) or 37°C (green line) for 2 h before analysis. This result was represented from one of three independent experiments.
